# CONDITIONAL DELETION OF TBX4/5 CAUSES PULMONARY HYPERTENSION THROUGH DISRUPTED EPITHELIAL, VASCULAR, AND SMOOTH MUSCLE DEVELOPMENT

**DOI:** 10.51894/001c.123041

**Published:** 2024-08-30

**Authors:** Kaylie Chiles

**Affiliations:** 1 College of Osteopathic Medicine Michigan State University

## 63

### INTRODUCTION

T-BOX transcription factor 4 (*TBX4*) mutations cause lung disorders including neonatal pulmonary arterial hypertension (PAH), alveolar capillary dysplasia, and acinar dysplasia; however, it remains unclear how loss of *TBX4* contributes to these clinical conditions. *TBX4* and *TBX5* are homologous genes key for organogenesis, and mouse models with whole-body deletion of these genes are embryonic lethal. Conditional deletion of these genes suggests a role in fetal lung development; however, neonatal and postnatal roles of these T-BOX genes have not been studied. A mouse model with complete deletion of both *TBX4* and *TBX5* that is viable at birth will provide insights into developmental origins of epithelial and vascular abnormalities in neonatal lung diseases.

### METHODS

We use Tbx4-Lung Mesenchyme Specific Enhancer (LME) Cre recombinase to conditionally delete *TBX4* and *TBX5*. Using this model, we will determine how *TBX4* and *TBX5* regulate airway and vessel development during in utero and postnatal lung development.

### OBJECTIVES/HYPOTHESIS

We are studying how *TBX4* and *TBX5* instruct mesenchymal differentiation, epithelial branching, and vascular development during lung development. We hypothesize that lung-specific deletion of *TBX4* and *TBX5* will result in abnormal mesenchymal progenitor cell fate specification and differentiation, causing disrupted lung branching, abnormal vascularization, and impaired airway function.

### RESULTS

We observe that combinatorial loss of *TBX4* and *TBX5* yields diminished epithelial branching, ectopic and excess smooth muscle cell (SMC) formation, increased fetal lung vessel density resulting in undersized lungs in fetal, early postnatal, and adult mice. Further, adult mice with T-BOX gene depletion show ectopic mesothelial SMCs and signs of PAH and right ventricle hypertrophy using echocardiography.

### DISCUSSION/CONCLUSIONS

Excessive SMCs that are needed for both airway and vessel function may explain how loss of T-BOX genes contributes to PAH. Since ectopic SMCs are observed in fetal stages, *TBX4* and *TBX5* could regulate allocation of mesenchymal progenitor pool to SMC fate. Postnatally, excess SMC accumulation may indicate a role for *TBX4* and *TBX5* in fibrosis. Clarifying the function of *TBX4* and *TBX5* in lung epithelial, vascular, and smooth muscle development will provide clinically relevant insights into the role of T-BOX genes in PAH, alveolar capillary dysplasia, and acinar dysplasia and the developmental origins of these diseases.

Research supported by Jean P. Schultz Biomedical Research Endowment Award.

